# *-DCC: A platform to collect, annotate, and explore a large variety of sequencing experiments

**DOI:** 10.1093/gigascience/giaa024

**Published:** 2020-03-14

**Authors:** Matthias Hörtenhuber, Abdul K Mukarram, Marcus H Stoiber, James B Brown, Carsten O Daub

**Affiliations:** 1 Department of Biosciences and Nutrition, Karolinska Institutet, NEO, Huddinge SE-141 83, Sweden; 2 Department of Statistics, University of California Berkeley, 367 Evans Hall,, Berkeley, CA 94720, USA; 3 Science for Life Laboratory, Tomtebodavägen 23A, Solna SE-171 65, Sweden

**Keywords:** databases, sequencing experiments, sample annotation, sequencing data annotation

## Abstract

**Background:**

Over the past few years the variety of experimental designs and protocols for sequencing experiments increased greatly. To ensure the wide usability of the produced data beyond an individual project, rich and systematic annotation of the underlying experiments is crucial.

**Findings:**

We first developed an annotation structure that captures the overall experimental design as well as the relevant details of the steps from the biological sample to the library preparation, the sequencing procedure, and the sequencing and processed files. Through various design features, such as controlled vocabularies and different field requirements, we ensured a high annotation quality, comparability, and ease of annotation. The structure can be easily adapted to a large variety of species. We then implemented the annotation strategy in a user-hosted web platform with data import, query, and export functionality.

**Conclusions:**

We present here an annotation structure and user-hosted platform for sequencing experiment data, suitable for lab-internal documentation, collaborations, and large-scale annotation efforts.

## Findings

### Background

Recent years showed a great increase in sequencing data quantity, as well as in the variety of experimental designs and sequencing techniques used [1]. This leads to great opportunities for addressing and complementing research questions with already available sequencing data. A crucial aspect here is to be able to first find the appropriate data and then to use them in harmony with the underlying conducted biological experiments [2]. The systematic description of the available sequencing data together with the description of the underlying biological experiments and sample details are a critical prerequisite.

The open science concept requires publication of the sequencing data alongside the scientific results [3]. Sequencing databases such as the SRA [4] or the Gene Expression Omnibus (GEO) [5] collect and open raw or processed sequencing data to the community and provide identifiers to connect data to scientific publications. The sequencing data are accompanied by an often minimalistic high-level description of experiments, samples, and technologies used [6].

Genome annotation projects including ENCODE [7], ModENCODE [8], and FANTOM [9] describe experimental aspects more systematically and with a greater level of detail. Together with the provided sophisticated query and export functionalities, this enables consistent processing of sequencing data and further allows direct comparison between all data within the projects. At the same time, substantial human resources are required for such data annotation and curation [10]. However, the underlying technical solutions were specific for each of these projects and were not designed to be generalizable to other contexts because of lack of access and documentation and design approach of the source code.

Here, we present a strategy to systematically annotate sequencing data together with their corresponding biological experiments. We implemented the strategy as a web server–based platform with a user-friendly interface allowing data collection and decentralized data annotation. This Data Coordination Center (*-DCC) constitutes a generic and flexible framework designed to be adaptable to hold data from various types and species. The user interface for uploading data was inspired by the SRA Submission Portal Wizard [11]. The query and export interface was designed similar to the ENCODE DCC data interface [12].

The *-DCC presented here is suitable for large-scale annotation efforts such as the DANIO-CODE genome annotation project [13]. Sequencing data management for 1 laboratory can be facilitated by the DCC with the added benefit of allowing sharing of selected data with various other laboratories.

### Annotation structure

**Figure 1:**
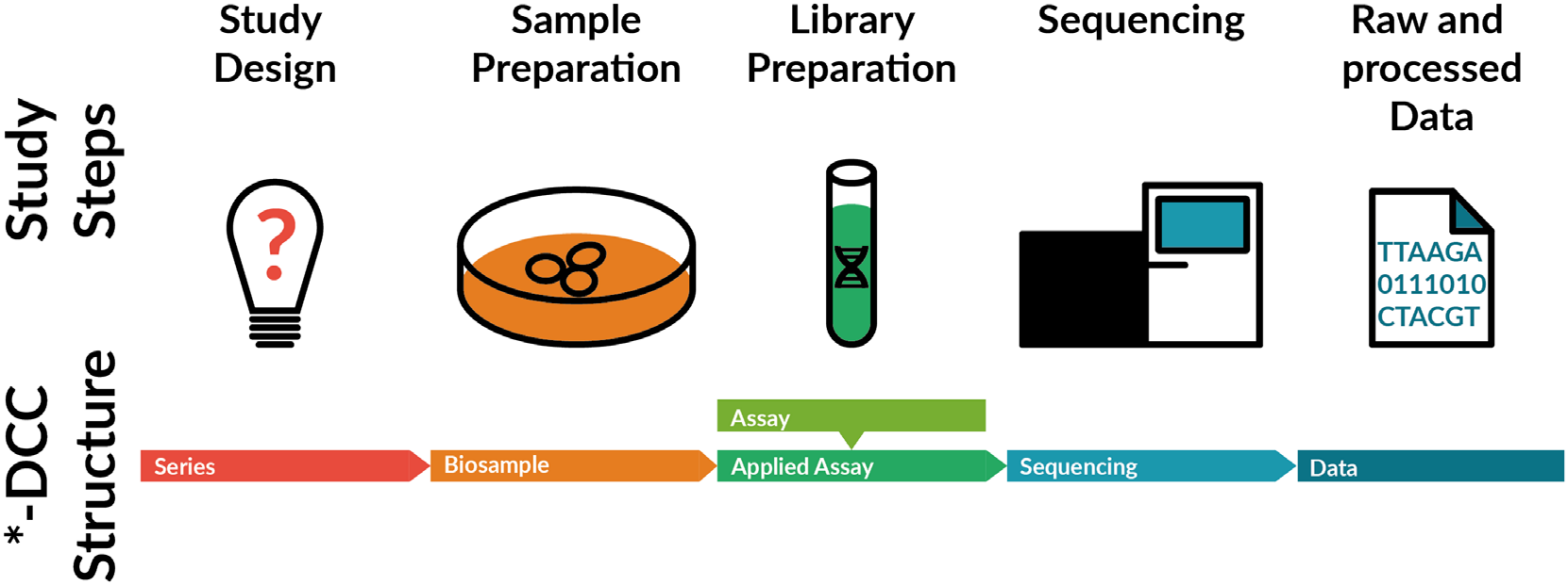
Overview of the *-DCC annotation structure. The *-DCC structure was designed to capture all study steps necessary for downstream analysis and groups information in sections in parallel to the study steps that a generic sequencing experiment is composed of.

The description of data is overall guided by the design of the conducted experiments and the corresponding experimental workflow (Fig. 1).

All experiments of 1 study targeting the same research question are collected under 1 common series object, which also contains the description of the overall purpose of the experiments. As an example, a case-control study with a number of animals with genetic mutation and their corresponding wild-type controls inspecting respective gene expression and histone marks would constitute a typical series.

The next level in the annotation is the description of the biosample, e.g., the age or developmental stage of the animals, genetic background, or the anatomical origin of the samples. This includes labelling biosamples as biological controls or biological replicates.

The assay level captures the type of assay and the library protocol details, such as RNA sequencing, chromatin immunoprecipitation sequencing (ChIP-Seq), or the immunoprecipitation targets used. It is independent of the aforementioned levels, allowing the same instance of an assay to be used in different series instances. The assays are in practice often identical with sequencing library preparation kits and are applied to the biological samples, resulting in applied assay objects. On this level, technical controls and replicates are labelled as such. Following the experimental workflow, the applied assays are sequenced using a specific platform and instrument with corresponding settings, all of which is captured in the sequencing level.

Finally, the sequencing files are the immediate results of the sequencing process together with corresponding files resulting from data processing. These files are described on the data level, which can also include additional information, e.g., about the genome version or the processing pipeline used.

Where applicable, we limit the annotation to a set of predefined terms. This aspect on the one hand unifies the metadata; on the other hand it guides the annotators to find the most appropriate terms and ensures a high level of annotation consistency. The controlled vocabulary constitutes the most species-specific part of our platform and might require adaptation to the species of interest.

Our annotation strategy requires certain terms to be provided by the annotator during the annotation process, e.g., whether the experimental design is based on a case-control or a survey layout. Other terms are only required under certain circumstances, e.g., the assay target has to be provided only for ChIP-seq and other immunoprecipitation assays. A third category is the optional fields that allow further information to be entered and queried in a structured way, e.g., the maximal read length of a sequence.

### Upload of data and annotations

To give a better insight into the specifics of uploading data to *-DCC, we compared the upload workflows between SRA and *-DCC. SRA provides an interactive annotation platform called Submission Portal Wizard and uses Microsoft Excel files or web forms for data input. Similarly, *-DCC provides a CSV-based and a web form–based submission option. To compare the 2 platforms adequately, we went through the 2 form-based approaches for a typical zebrafish sequencing experiment as an example. The SRA covers a wider scope of experiments and data sources, e.g., metagenome studies and pathogen studies, compared with *-DCC. Therefore, we discuss only the relevant matching options in SRA.

After login, the SRA Submission Portal Wizard starts by requesting information about the submitter. This information is entered indirectly in the *-DCC by specifying a laboratory for the Biosample, Assay, and Sequencing sections and by the information entered during user registration about the currently logged-in user. Besides being logged in on *-DCC, only users with the annotator role have permission to upload annotations and data.

The General Info step on the SRA platform asks for already created bioProject and bioSample instances related to this upload, as well as a publication date for the uploaded data to go public. In the *-DCC form, a PubMed and a GEO ID can be provided for similar purposes. Also, the series can be set as public, i.e., visible to every user of the platform, or to only be visible to the currently operating user. Such private datasets can later be opened to the public.

**Figure 2:**
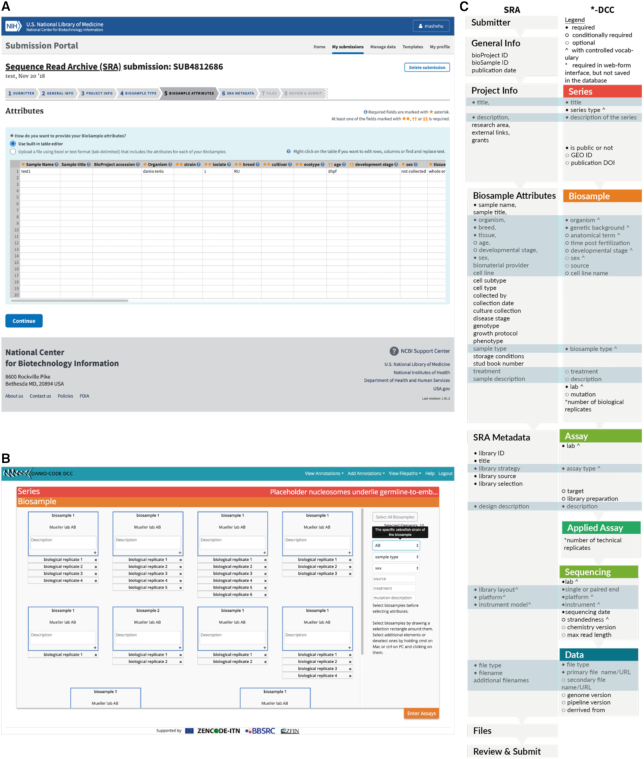
Comparison of the annotation upload between the SRA Submission Portal Wizard and the *-DCC web-form. **A**, Screenshot of the Biosample Attributes annotation section of the SRA. **B**, Screenshot of the Biosample annotation section of the *-DCC. **C**, Comparison of the fields used in the SRA (left column) and *-DCC (right column). Shared terms are horizontally aligned and highlighted in blue. See the supplementary material for the definition of each field. The field “number of biological/technical replicates” is only required in web-form interface and is not directly represented in the database. Screenshot from the SRA Submission Portal Wizard was taken by the author on July 5, 2019 from https://submit.ncbi.nlm.nih.gov/subs/sra/SUB5927179/attributes.

The next step collects the biological details on both platforms. In contrast to the SRA, the majority of these fields are connected to a controlled vocabulary on *-DCC. On both platforms some annotation fields are required to be filled while others are optional. A detailed comparison between the different terms is provided in Fig. 2C.

The SRA Metadata step of the Submission Portal Wizard corresponds to 4 distinct steps in *-DCC, which are Assay, Applied Assay, Sequencing, and Data. These steps contain details about the library preparation, sequencing instrument, and the data files. Both platforms provide a controlled vocabulary for several fields of this section via dropdown menus. The *-DCC allows the capture of additional information about the sequencing settings and allows the same assay to be used in a different series entry.

In the final 2 steps of the Submission Portal Wizard, the uploader provides the files either locally, via FTP preloads, or via Amazon S3 buckets. Afterwards, the whole annotation is submitted. On the *-DCC, the file upload takes place in the Data section, by providing a URL to a web-accessible file or the file path on the DCC server for previously uploaded files. Users are recommended to stay on the upload page until a confirmation of the successful upload appears, but the upload will continue even if the page is closed. Depending on the file sizes and the internet connection of both the *DCC-server and the annotator, the file upload can last a few minutes up to hours (see also Methods).

### Query and export of data and annotations

**Figure 3:**
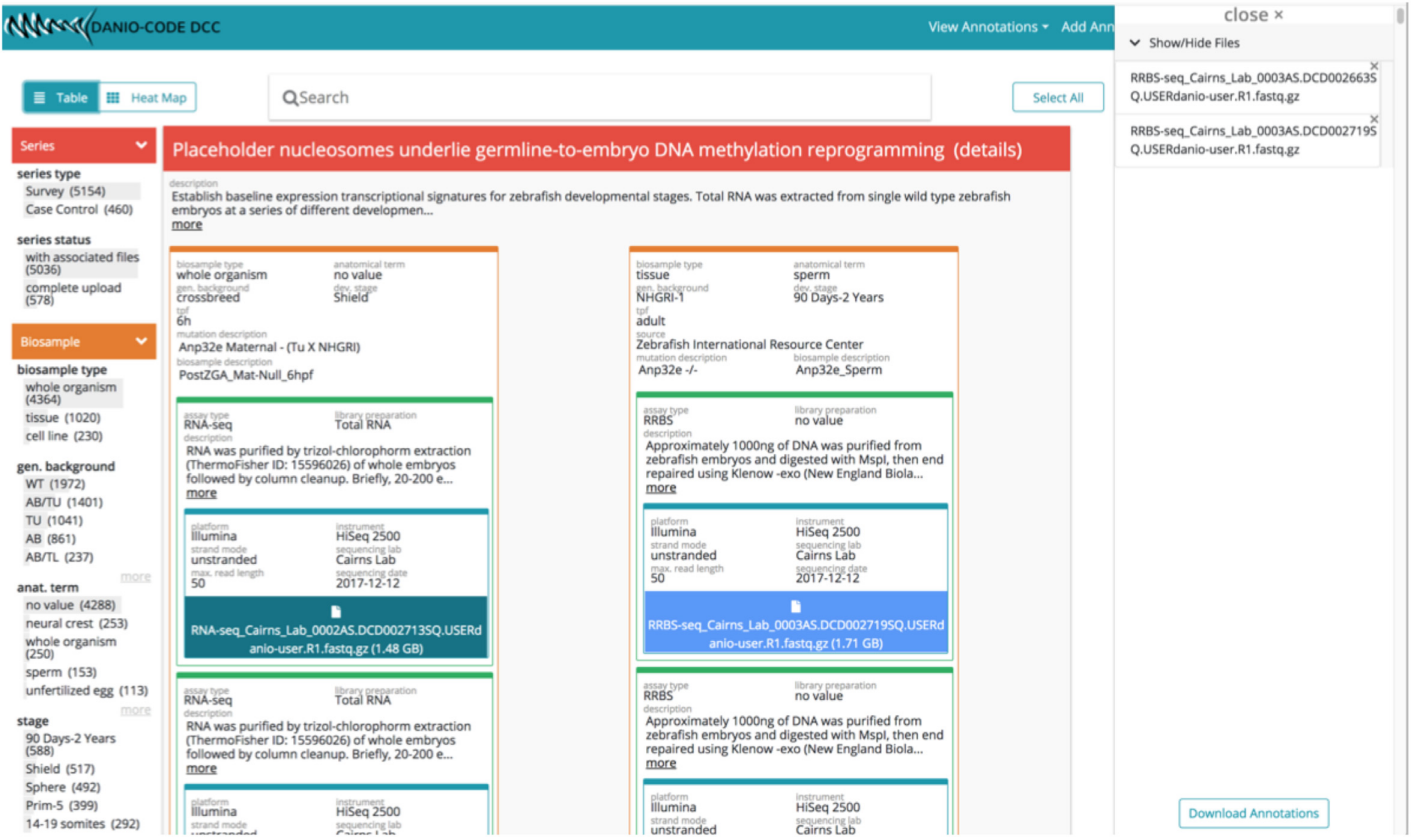
User interface of the DCC implementation for the DANIO-CODE consortium. The data export view with filter facets (left sidebar) and datasets selected for export (right sidebar).

In order to query and export data and their annotations, *-DCC provides a table view with filter options (Fig. 3) as well as an interactive heat map, similar to the matrix view in the ENCODE DCC. Together with the annotation structure, these data views allow pooling of different series based on a combination of shared annotation terms, e.g., based on the same assay or the same developmental stage, by clicking on the relevant terms in the left sidebar. Similar to the ENCODE DCC, the *-DCC sidebar indicates the number of occurrences of each term in the present table. This enables a quick identification of complementary datasets for later integrative analysis.

*-DCC allows the download of sequencing files, as well as their accompanying annotations. The annotation file can then be used with data-processing pipelines to select processing parameters based on the annotations. This might make it a suitable platform for a consortium or large-scale studies. The DANIO-CODE consortium uses *-DCC to collect and annotate zebrafish sequencing data [14].

### Limitations

*-DCC was not designed as a laboratory information management system (LIMS) and therefore was not built to capture every detail of an experiment. We limited the platform to aspects necessary for downstream analysis, and integrative studies are covered. For the same reasons, *-DCC does not provide any API for automated annotation and data uploads.

Furthermore, the main goal for *-DCC is to capture genomics laboratory experiments and as a result was not designed to capture, for example, the collection locations of metagenomics studies.

## Methods

*-DCC was implemented as a Django 1.11 app with an underlying PostgreSQL database and a JavaScript-supported front end. We rely on Django's user management framework with 4 different roles: guest, viewer, annotator, and administrator. Guests are users who are not logged in and have restricted access to sites on the platform. Logged-in users, termed “viewers,” can have additional access to items such as, e.g., datasets that have not been set to public (not activated in the demo set-up). Annotators are given wider access including to the tools for the upload of annotations and data. The administrator can access the Django administration page to make changes to the database, fix broken uploads, and handle user roles.

The file upload occurs asynchronously using the Django file interface and ajax calls. The platform can therefore handle multiple uploads at the same time. Depending on the file sizes and the internet connection of both the *-DCC server and the annotator, the file upload can last from a few minutes up to hours. For larger files (>10 GB), we recommend preloading them on the server to speed up the process. Broken uploads have to be taken care of by the administrator manually.

Unit tests based on Django's test framework, as well as end-to-end tests via cypress, are available. A Docker container and installation instructions are available.

## Availability of Supporting Source Code and Requirements

- Project name: *-DCC
- Project home page: https://gitlab.com/danio-code/public/dcc
- Operating systems: Linux (tested on Red Hat Enterprise Linux Server 7.4 and CentOS Linux release 7.6.1810 [Core])
- Programming languages: Python 2.7, JavaScript ECMAScript 2018
- Other requirements: PostgreSQL 9.2.23, Node 12.2.0
- License: MIT
- RRID:SCR_016544

The source code is available under the MIT license at https://gitlab.com/danio-code/public/dcc. Unit tests as well as end-to-end tests are available.

A Docker container is available in the repository for testing and deployment; see dcc.readthedocs.io for further instructions and code documentation.

A demo implementation is running on http://dcc-demo.daublab.org/ with username “annotator” and password “annotator” to have annotator user rights.

## Supplementary Material

giaa024_GIGA-D-19-00400_Original_Submission

giaa024_GIGA-D-19-00400_Revision_1

giaa024_GIGA-D-19-00400_Revision_2

giaa024_Response_to_Reviewer_Comments_Original_Submission

giaa024_Response_to_Reviewer_Comments_Revision_1

giaa024_Reviewer_1_Report_Original_Submission Christophe Trefois -- 12/20/2019 Reviewed

## Availability of Supporting Data and Materials

Snapshots of the code are available in the GigaScience GigaDB repository [15].

## Abbreviations

API: Application Programming Interface; ChIP-Seq: chromatin immunoprecipitation sequencing; CSV: comma-separated values; DCC: Data Coordination Center; ENCODE: Encyclopedia of DNA Elements; GEO: Gene Expression Omnibus; LIMS: laboratory information management system; SRA: Sequence Read Archive.
