# Supplementary material for: *-DCC: A platform to collect, annotate, and explore a large variety of sequencing experiments
Source: Gigascience. 2020 Mar 14;9(3):giaa024. doi: 10.1093/gigascience/giaa024 (PMC7069921; doi:10.1093/gigascience/giaa024)
Supplement: giaa024_GIGA-D-19-00400_Revision_2 [file giaa024_giga-d-19-00400_revision_2.pdf]

GigaScience

*-DCC: A platform to collect, annotate and explore a large variety of sequencing experiments

--Manuscript Draft--

**Manuscript Number:**	GIGA-D-19-00400R2
**Full Title:**	*-DCC: A platform to collect, annotate and explore a large variety of sequencing experiments
**Article Type:**	Research
**Funding Information:**	H2020 Marie Skłodowska-Curie Actions (643062) — Dr Carsten Oliver Daub
**Abstract:**	Background: Over the last few years the variety of experimental designs and protocols for sequencing experiments increased greatly. To assure the wide usability of the produced data beyond an individual project, rich and systematic annotation of the underlying experiments is of outstanding importance.<br>Findings: We first developed an annotation structure which captures the overall experimental design as well as the relevant details of the steps from the biological sample to the library preparation, the sequencing procedure and to the sequencing and processed files. Through various design features, such as controlled vocabularies and different field requirements, we ensured a high annotation quality, comparability and ease of annotation. The structure can be easily adapted to a large variety of species. We then implemented the annotation strategy in a user-hosted web-platform with data import, query and export functionality.<br>Conclusions: We present here an annotation structure and user-hosted platform for sequencing experiment data, suitable for lab-internal documentation, collaborations and large-scale annotation efforts.
**Corresponding Author:**	Carsten Oliver Daub, Ph.D.<br>Karolinska Institutet<br>Huddinge, Stockholm SWEDEN
**Corresponding Author Secondary Information:**	
**Corresponding Author's Institution:**	Karolinska Institutet
**Corresponding Author's Secondary Institution:**	
**First Author:**	Matthias Hörtenhuber
**First Author Secondary Information:**	
**Order of Authors:**	Matthias Hörtenhuber
	Abdul Kadir Mukarram
	Marcus Stoiber, PhD
	James B Brown, PhD
	Carsten Oliver Daub, Ph.D.
**Order of Authors Secondary Information:**	
**Response to Reviewers:**	Dear Scott,<br><br>We added an Availability of Supporting Data section in the paper and now cite the GigaDB snapshot (reference [14]).<br><br>Best,<br>Carsten
**Additional Information:**	
**Question**	**Response**

Powered by Editorial Manager® and ProduXion Manager® from Aries Systems Corporation

Are you submitting this manuscript to a special series or article collection?	No
**Experimental design and statistics**<br><br>Full details of the experimental design and statistical methods used should be given in the Methods section, as detailed in our Minimum Standards Reporting Checklist. Information essential to interpreting the data presented should be made available in the figure legends.<br><br>Have you included all the information requested in your manuscript?	Yes
**Resources**<br><br>A description of all resources used, including antibodies, cell lines, animals and software tools, with enough information to allow them to be uniquely identified, should be included in the Methods section. Authors are strongly encouraged to cite Research Resource Identifiers (RRIDs) for antibodies, model organisms and tools, where possible.<br><br>Have you included the information requested as detailed in our Minimum Standards Reporting Checklist?	Yes
**Availability of data and materials**<br><br>All datasets and code on which the conclusions of the paper rely must be either included in your submission or deposited in publicly available repositories (where available and ethically appropriate), referencing such data using a unique identifier in the references and in the "Availability of Data and Materials" section of your manuscript.<br><br>Have you have met the above requirement as detailed in our Minimum	Yes

Standards Reporting Checklist?	

Powered by Editorial Manager® and ProduXion Manager® from Aries Systems Corporation

Manuscript

Click here to access/download;Manuscript;Giga Science DCC paper 2020-02-19.pdf

Title page

Title: *-DCC: A platform to collect, annotate and explore a large variety of sequencing experiments

Authors: Matthias Hörtenhuber[1](matthias.hortenhuber@ki.se), Abdul K. Mukarram[1] (abdul.kadir.mukarram@ki.se), Marcus H. Stoiber[2] (marcus.stoiber@gmail.com), James B. Brown[2] (bbrown@lbl.gov), Carsten O. Daub[1,3]* (carsten.daub@ki.se)
[1]Department of Biosciences and Nutrition, Karolinska Institutet, Huddinge, SE-141 83, Sweden.
[2]Department of Statistics, University of California Berkeley, Berkeley, California 94720, USA
[3] Science for Life Laboratory, Stockholm, Sweden
* corresponding author

Abstract

Background: Over the last few years the variety of experimental designs and protocols for sequencing experiments increased greatly. To assure the wide usability of the produced data beyond an individual project, rich and systematic annotation of the underlying experiments is of outstanding importance.
Findings: We first developed an annotation structure which captures the overall experimental design as well as the relevant details of the steps from the biological sample to the library preparation, the sequencing procedure and to the sequencing and processed files. Through various design features, such as controlled vocabularies and different field requirements, we ensured a high annotation quality, comparability and ease of annotation. The structure can be easily adapted to a large variety of species. We then implemented the annotation strategy in a user-hosted web-platform with data import, query and export functionality.
Conclusions: We present here an annotation structure and user-hosted platform for sequencing experiment data, suitable for lab-internal documentation, collaborations and large-scale annotation efforts.

Keywords

databases, sequencing experiments, sample annotation, sequencing data annotation

Findings

Background

Recent years showed a great increase in sequencing data quantity as well as in a variety of employed experimental designs and sequencing techniques [1]. This leads

to great opportunities for addressing and complementing research questions with already available sequencing data. A crucial aspect here is to be able to first find the appropriate data and then to utilize them in harmony with the underlying conducted biological experiments [2]. The systematic description of the available sequencing data together with the description of the underlying biological experiments and sample details are a critical prerequisite.

The open science concept requires publication of the sequencing data alongside the scientific results [3]. Sequencing databases such as the Sequence Read Archive (SRA) [4] or the Gene Expression Omnibus (GEO) [5] collect and open raw or processed sequencing data to the community and provide identifiers to connect data to scientific publications. The sequencing data are accompanied by an often minimalistic high-level description of experiments, samples and technologies employed [6].

Genome annotation projects including ENCODE [7], ModENCODE [8] and FANTOM [9] describe experimental aspects more systematically and with a greater level of detail. Together with the provided sophisticated query and export functionalities, this enables consistent processing of sequencing data and further allows direct comparison between all data within the projects. At the same time, significant human resources are required for such data annotation and curation [10]. However, the underlying technical solutions were specific for each of these projects and were not designed to be generalizable to other contexts, because of lack of access and documentation and design approach of the source code.

Here, we present a strategy to systematically annotate sequencing data together with their corresponding biological experiments. We implemented the strategy as a webserver-based platform with a user-friendly interface allowing data collection and decentralized data annotation. This Data Coordination Center (*-DCC) constitutes a generic and flexible framework designed to be adaptable to hold data from various types and species. The user interface for uploading data was inspired by the SRA Submission Portal Wizard [11]. The query and export interface was designed similar to the ENCODE DCC data interface [12].
The *-DCC presented here is suitable for large-scale annotation efforts such as the DANIO-CODE genome annotation project [13]. Sequencing data management for one lab can be facilitated by the DCC with the added benefit of allowing sharing of selected data with various other labs.

Annotation Structure

The description of data is overall guided by the design of the conducted experiments and the corresponding experimental workflow (Figure 1).

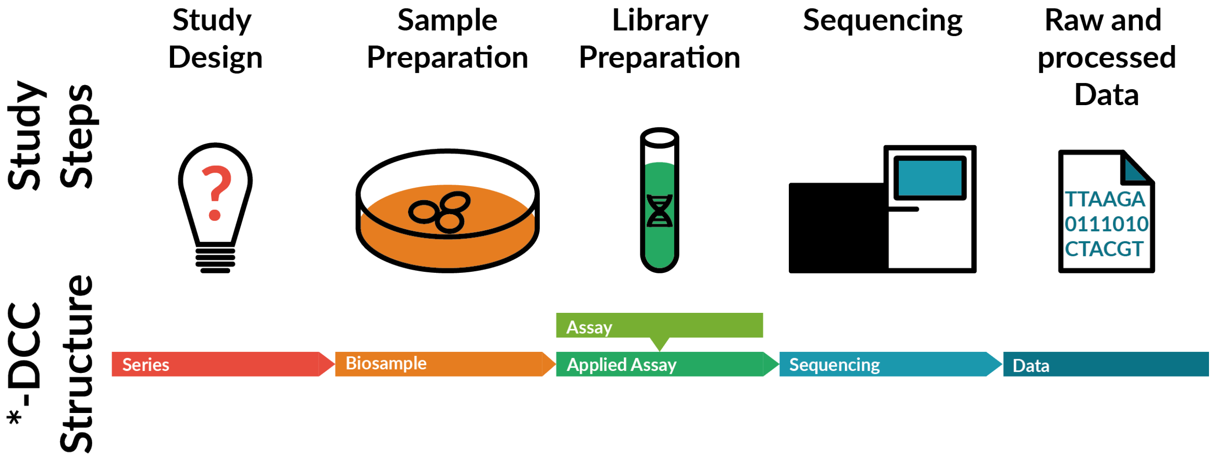

Figure 1. Overview of the *-DCC annotation structure. The *-DCC structure was designed to capture all study steps necessary for downstream analysis and groups information in sections in parallel to the study steps a generic sequencing experiment is composed of.

All experiments of one study targeting the same research question are collected under one common series object, which also contains the description of the overall purpose of the experiments. As an example, a case-control-study with a number of animals with genetic mutation and their corresponding wild-type controls inspecting respective gene expression and histone marks would constitute a typical series.
The next level in the annotation is the description of the biosample, for example the age or developmental stage of the animals, genetic background or the anatomical origin of the samples. This includes labelling biosamples as biological controls or biological replicates.
The assay level captures the type of assay and the library protocol details, such as RNA-Seq, ChIP-Seq or the used immunoprecipitation targets. It is independent of the above-mentioned levels, allowing to use the same instance of an assay in different series instances. The assays are in practice often identical with sequencing library preparation kits and are applied to the biological samples resulting in applied assay objects. On this level, technical controls and replicates are labelled as such.
Following the experimental workflow, the applied assays are sequenced using a specific platform and instrument with corresponding settings, all of which is captured in the sequencing level.
Finally, the sequencing files are the immediate results of the sequencing process together with corresponding files resulting from data processing. These files are described on the data level, which can also include additional information for example about the genome version or the employed processing pipeline.

Where applicable, we limit the annotation to a set of predefined terms. This aspect unifies on the one hand the metadata, on the other hand it guides the annotators to find the most appropriate terms and ensures a high level of annotation consistency. The controlled vocabulary constitutes the most species-specific part of our platform and might require adaptation to the species of interest.

Our annotation strategy requires certain terms to be provided by the annotator during the annotation process, for example whether the experimental design is based on a case-control or a survey layout. Other terms are only required under certain circumstances, for example the assay target has to be provided only for ChIP-seq and other immunoprecipitation assays. A third category are the optional fields that allow further information to be entered and queried in a structured way, for example the maximal read length of a sequence.

Upload of data and annotations

To give a better insight into the specifics of uploading data to *-DCC, we compared the upload workflows between SRA and *-DCC. SRA provides an interactive annotation platform called Submission Portal Wizard and uses Microsoft Excel files or web forms for data input. Similarly, *-DCC provides a csv-based and a web form-based submission option. In order to compare the two platforms adequately, we went through the two form-based approaches for a typical zebrafish sequencing experiment as an example. The SRA covers a wider scope of experiments and data sources, e.g. metagenome studies and pathogen studies, compared to *-DCC. Therefore, we discuss only the relevant matching options in SRA.

After login, the SRA Submission Portal Wizard starts by requesting information about the submitter. This information is entered indirectly in the *-DCC by specifying a lab for the Biosample, Assay and Sequencing sections and by the information entered during user registration about the currently logged-in user. Besides being logged-in on *-DCC, only users with the annotator role have the permission to upload annotations and data.

The General Info step on the SRA platform asks for already created bioProject and bioSample instances related to this upload as well as a publication date for the uploaded data to go public. In the *-DCC form, a PubMed and a GEO ID can be provided for similar purposes. Also, the series can be set as public, that is visible to every user of the platform, or to only be visible to the currently operating user. Such private datasets can later be opened to the public.

The next step collects the biological details on both platforms. In contrast to the SRA, the majority of these fields are connected to a controlled vocabulary on *-DCC. On both platforms some annotation fields are required to be filled while others are only optional. A detailed comparison between the different terms are provided in Figure 2C.

The SRA Metadata step of the Submission Portal Wizard corresponds to four distinct steps in *-DCC, which are Assay, Applied Assay, Sequencing and Data. These steps contain details about the library preparation, sequencing instrument and the data files. Both platforms provide a controlled vocabulary for several fields of this section via drop down menus. The *-DCC allows to hold additional information about the sequencing settings and allows the same assay to be used in a different series entry.

In the final two steps of the Submission Portal Wizard, the uploader provides the files either locally, via FTP preloads or via Amazon S3 buckets. Afterwards, the whole annotation is submitted. On the *-DCC, the file upload takes place in the Data section, by providing a URL to a web-accessible file or the file path on the DCC server for previously uploaded files. Users are recommended to stay on the upload page until a confirmation of the successful upload appears, but the upload will continue even if the page is closed. Depending on the

file sizes and the internet connection of both the *DCC-server and the annotator, the file upload can last a few minutes up to hours (see also Methods).

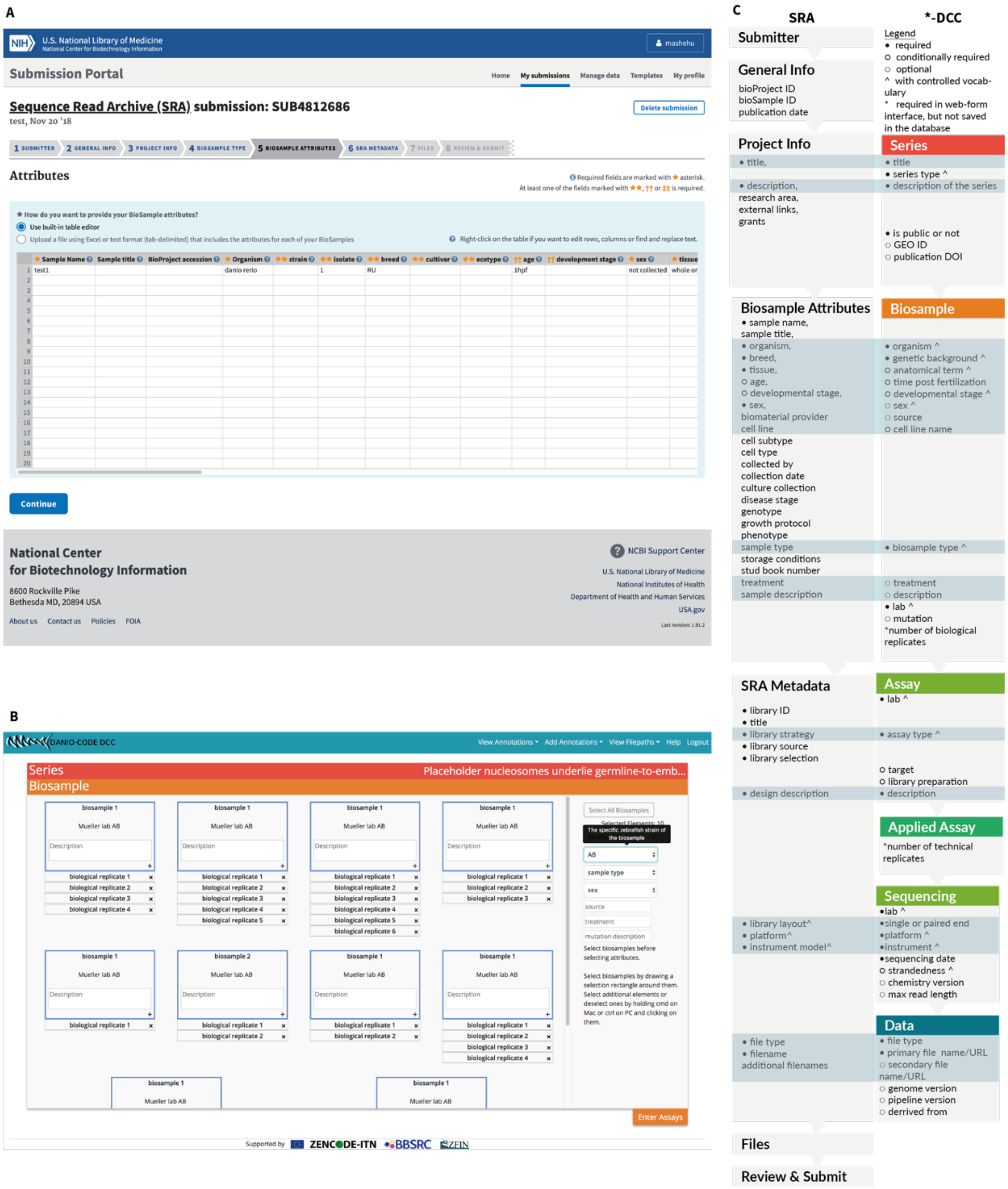

Figure 2. Comparison of the annotation upload between the SRA Submission Portal Wizard and the *-DCC web-form. **A)** Screenshot of the Biosample Attributes annotation section of the SRA. **B)** Screenshot of the Biosample annotation section of the *-DCC. **C)** Comparison of the fields used in the SRA (left column) and *-DCC (right column). Shared terms are horizontally aligned and highlighted in blue. See the supplementary material for the definition of each field. The fields “number of biological/technical replicates” are only required in web-form interface and are not directly represented in the database.

Query and export of data and annotations

In order to query and export data and their annotations, *-DCC provides a table view with filter options (Figure 3) as well as an interactive heat map, similar to the matrix view in the ENCODE DCC. Together with the annotation structure, these data views allow pooling of different series based on a combination of shared annotation terms for example based on the same assay or the same developmental stage, by clicking on the relevant terms in the left sidebar. Similar to the ENCODE DCC, the *-DCC sidebar indicates the number of occurrences of each term in the current table. This enables a quick identification of complementary data sets for later integrative analysis.

The *-DCC allows the download of sequencing files, as well as their accompanying annotations. The annotation file can then be used with data processing pipelines to select processing parameters based on the annotations. This might make it a suitable platform for a consortium or large-scale studies. The DANIO-CODE consortium uses *-DCC to collect and annotate zebrafish sequencing data (available at danio-code.zfin.org).

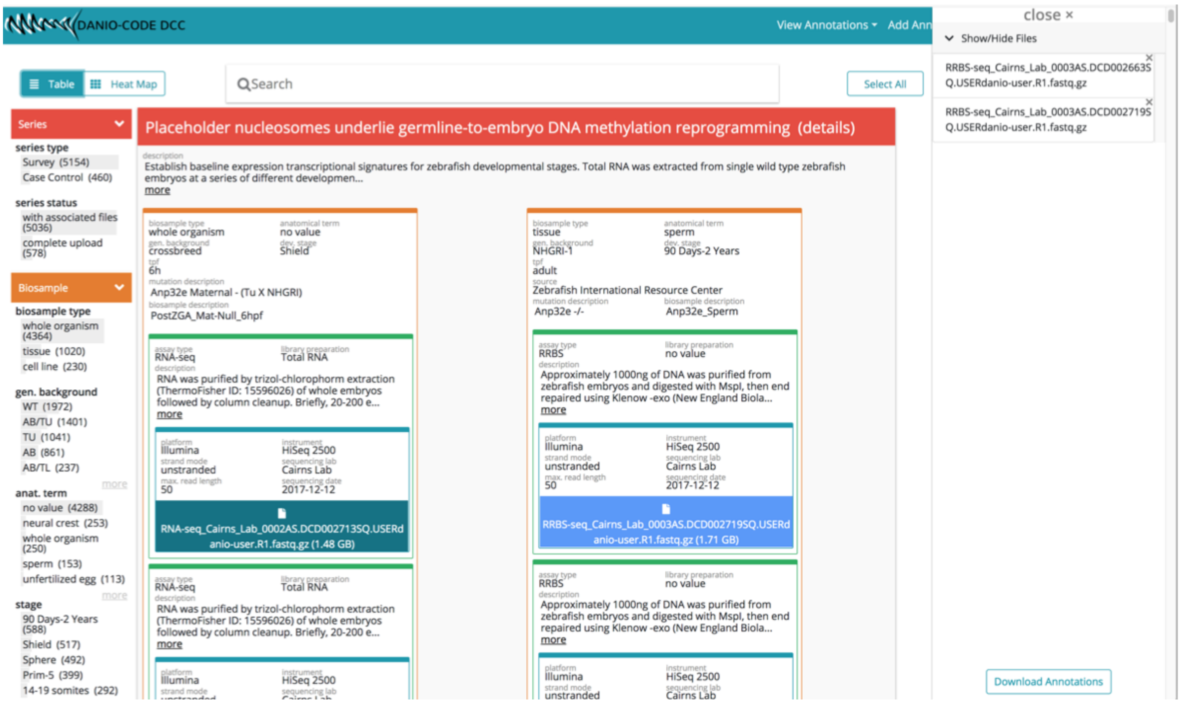

Figure 3. User interface of the DCC implementation for the DANIO-CODE consortium. The data export view with filter facets (left sidebar) and data sets selected for export (right sidebar).

Limitations

*-DCC was not designed as a Laboratory information management system (LIMS) and therefore was not built to capture every detail of an experiment. We limited the platform to aspects necessary for down-stream analysis and integrative studies are covered. For the same reasons, *-DCC does not provide any API for automated annotation and data uploads. Furthermore, the main goal for *-DCC is to capture genomics lab experiments and as a result wasn't designed to capture for example the collection locations of metagenomics studies.

Methods

*-DCC was implemented as a Django 1.11 app with an underlying PostgreSQL database and a JavaScript-supported frontend. We rely on Django's user management framework with four different roles: guest, viewer, annotator and admin. Guests are users who are not logged in and have restricted access to sites on the platform. Logged in users, termed viewers, can have additional access such as for example data sets, which have not been set to public (not activated in the demo setup). Annotators are given wider access including to the tools for the upload of annotations and data. The admin can access the Django admin page to make changes to the database, fix broken uploads and handle user roles.

The file-upload occurs asynchronously using the Django file interface and ajax calls. The platform can therefore handle multiple uploads at the same time. Depending on the file sizes and the internet connection of both the *-DCC-server and the annotator, the file upload can last from a few minutes up to hours. For larger files (>10GB), we recommend preloading them on the server to speed up the process. Broken uploads will have to be taken care of by the admin manually.

Unit tests based on Django's test framework as well as end-to-end tests via cypress are available. A docker container and installation instructions are available.

Availability of supporting source code and requirements

Availability and requirements

- Project name: *-DCC
- Project home page: https://gitlab.com/danio-code/public/dcc

- Operating systems: Linux (tested on Red Hat Enterprise Linux Server 7.4 and CentOS Linux release 7.6.1810 (Core))
- Programming languages: Python 2.7, JavaScript ECMAScript 2018
- Other requirements: PostgreSQL 9.2.23, Node 12.2.0
- License: MIT
- SciCrunch RRID: SCR_016544

The source code is available under the MIT license at https://gitlab.com/danio-code/public/dcc. Unit tests as well as end-to-end tests are available.

A docker container is available in the repository for testing and deployment, see dcc.readthedocs.io for further instructions and code documentation.

A demo implementation is running on http://dcc-demo.daublab.org/ with username "annotator" and password "annotator" to have annotator user rights.

Availability of Supporting Data

Snapshots of the code are available in the GigaScience GigaDB repository [14].

Declarations

List of abbreviations

DCC=Data Coordination Center
SRA=Sequence Read Archive
LIMS=Laboratory information management system

Ethics approval and consent to participate

Not applicable

Consent for publication

Not applicable

Competing interests

The authors declare that they have no competing interests.

Funding

This project has received funding from the European Union’s Horizon 2020 research and innovation programme under the Marie Skłodowska-Curie grant agreement No 643062, received by Carsten O. Daub.

Authors' contributions

MH, MHS, AKM, JBB and COD developed the annotation structure. MH, MS and AM programmed the platform. MH and COD wrote the manuscript.

Acknowledgements

We would like to thank ZFIN for help with ontology terms and for hosting the DANIO-CODE DCC.

References

1. Muir P, Li S, Lou S, Wang D, Spakowicz DJ, Salichos L, et al. The real cost of sequencing: scaling computation to keep pace with data generation. Genome Biol. 2016;17:53. doi:10.1186/s13059-016-0917-0.

2. Reddy TBK, Thomas AD, Stamatis D, Bertsch J, Isbandi M, Jansson J, et al. The Genomes OnLine Database (GOLD) v.5: a metadata management system based on a four level (meta)genome project classification. Nucleic Acids Res. 2015;43 Database issue:D1099-106. doi:10.1093/nar/gku950.

3. Vicente-Saez R, Martinez-Fuentes C. Open Science now: A systematic literature review for an integrated definition. J Bus Res. 2018;88:428–36. doi:10.1016/j.jbusres.2017.12.043.

4. Kodama Y, Shumway M, Leinonen R, International Nucleotide Sequence Database Collaboration. The Sequence Read Archive: explosive growth of sequencing data. Nucleic Acids Res. 2012;40 Database issue:D54-6. doi:10.1093/nar/gkr854.

5. Edgar R, Domrachev M, Lash AE. Gene Expression Omnibus: NCBI gene expression and hybridization array data repository. Nucleic Acids Res. 2002;30:207–10. doi:10.1093/nar/30.1.207.

6. Wang Z, Monteiro CD, Jagodnik KM, Fernandez NF, Gundersen GW, Rouillard AD, et al. Extraction and analysis of signatures from the Gene Expression Omnibus by the crowd. Nat Commun. 2016;7:12846. doi:10.1038/ncomms12846.

7. ENCODE Project Consortium. The ENCODE (encyclopedia of DNA elements) project. Science. 2004;306:636–40. doi:10.1126/science.1105136.

8. Celniker SE, Dillon LAL, Gerstein MB, Gunsalus KC, Henikoff S, Karpen GH, et al.

Unlocking the secrets of the genome. Nature. 2009;459:927–30. doi:10.1038/459927a.

9. Abugessaisa I, Shimoji H, Sahin S, Kondo A, Harshbarger J, Lizio M, et al. FANTOM5 transcriptome catalog of cellular states based on Semantic MediaWiki. Database (Oxford). 2016;2016. doi:10.1093/database/baw105.

10. Washington NL, Stinson EO, Perry MD, Ruzanov P, Contrino S, Smith R, et al. The modENCODE Data Coordination Center: lessons in harvesting comprehensive experimental details. Database (Oxford). 2011;2011:bar023. doi:10.1093/database/bar023.

11. Barrett T, Clark K, Gevorgyan R, Gorelenkov V, Gribov E, Karsch-Mizrachi I, et al. BioProject and BioSample databases at NCBI: facilitating capture and organization of metadata. Nucleic Acids Res. 2012;40 Database issue:D57-63. doi:10.1093/nar/gkr1163.

12. Sloan CA, Chan ET, Davidson JM, Malladi VS, Strattan JS, Hitz BC, et al. ENCODE data at the ENCODE portal. Nucleic Acids Res. 2016;44:D726-32. doi:10.1093/nar/gkv1160.

13. Tan H, Onichtchouk D, Winata C. DANIO-CODE: Toward an Encyclopedia of DNA Elements in Zebrafish. Zebrafish. 2016;13:54–60. doi:10.1089/zeb.2015.1179.

14. Hörtenhuber M; Mukarram AK; Stoiber M; Brown JB; Daub CO (2020): Supporting data for "*-DCC: A platform to collect, annotate and explore a large variety of sequencing experiments" GigaScience Database. http://dx.doi.org/10.5524/100714
