# Supplementary material for: *-DCC: A platform to collect, annotate, and explore a large variety of sequencing experiments
Source: Gigascience. 2020 Mar 14;9(3):giaa024. doi: 10.1093/gigascience/giaa024 (PMC7069921; doi:10.1093/gigascience/giaa024)
Supplement: giaa024_Response_to_Reviewer_Comments_Original_Submission [file giaa024_response_to_reviewer_comments_original_submission.pdf]

Author's Response To Reviewer Comments

Close

Comment:
The first instruction in the documentation is not bulletproof, as without a Gitlab account, the command fails. It is better to use https instead of git for this purpose. (Clone the git repository: $ git clone git@gitlab.com:danio-code/public/dcc.git)
After this, the docker image was successfully starting though.

Reply:
We changed the documentation accordingly.

Comment:
Outdated tools are used for developing the Django application. Django 1.11 is 2 years old and all related dependancies are probably riddled with security issues.
Granted, if the tool is only supposed to be used within a institution firewall this might be OK, but in general we recommend here to at least update to latest minor versions.

Reply:
Django 1.11 is under long term support, so it still receives regular security updates.

Comment:
Improvements:

Add CI/CD, Use SAST, Dependency Scanning or other features of Gitlab / Github to automatically keep updated with security functionalities.

Reply:
Thank you. We will consider the improvement suggestions for future updates.

Close
