# Supplementary material for: *-DCC: A platform to collect, annotate, and explore a large variety of sequencing experiments
Source: Gigascience. 2020 Mar 14;9(3):giaa024. doi: 10.1093/gigascience/giaa024 (PMC7069921; doi:10.1093/gigascience/giaa024)
Supplement: giaa024_Response_to_Reviewer_Comments_Revision_1 [file giaa024_response_to_reviewer_comments_revision_1.pdf]

Author's Response To Reviewer Comments

Close

Dear Scott,

We added an Availability of Supporting Data section in the paper and now cite the GigaDB snapshot (reference [14]).

Best,
Carsten

Close
